# One Certain Emmenagogue

**Published:** 1854-07

**Authors:** B. H. Washington

**Affiliations:** Hannibal, Mo.


### *One Certain Emmenagogue.*

BY B. H. WASHINGTON, OF HANNIBAL, MO.

DEAR DOCTOR:—In Prof. Eve's most interesting letters from Europe, just received by me, the following passages caught my attention. "Prof. Simpson (of Edinburgh,) has just operated upon a case, that of cupping directly the uterus for amenorrhœa. The fluid extracted was subjected to the microscope and exhibited blood corpuscles, as well as epithetial cells in the mucus. This method to bring on menstruation is resorted to when other means have failed, and is only adapted to a certain number of cases. Should the ordinary treatment fail, and galvanism produce no effect when applied as described, then as the dernier resort a long catheter is introduced into the womb, and a suction pump adapted to its external extremity. This is the direct cupping of the uterus, and is surely one certain emmenagogue." This plan, though not precisely like mine, is 'next kin' to it; and I therefore have taken my pen to invite attention again to the subject of dry-cupping on the spine as an emmenagogue, and hope the profession will be induced by the great authority of Prof. Simpson's name, to give my humble plan a trial. The difference between us is simply this, he recommends dry-cupping directly on the part affected; I recommend in all cases, dry-cupping over the origin of the nerves distributed to the affected part; and I confidently assert that no one will be at a loss to decide which plan is best, whether he tries dry-cupping to relieve colic, neuralgia, or as an emmenagogue; in every case the advantages of application over the origin of the nerves, instead of directly to the diseased part, will be found to predominate greatly.

I feel exceedingly anxious dry-cupping should be tried more extensively; nine years experience with it, has amply demonstrated the above fact, and the cases of extraordinary and prompt relief have been so numerous, that I cannot avoid thinking a very important remedial agent is too much overlooked. As you have many new subscribers, allow me to quote from an article I wrote in ''52.' "Some years since, I had a severe case of dysmenorrhea, of only five months duration, however. Feeling anxious to afford prompt relief, a celebrated physician was consulted, but not liking his plan, I concluded to try dry-cupping, as I had three weeks to go upon. The cups were applied every alternate night, the whole length of the spine, more strongly over the origin of the nerves distributed to the uterus; and the result was, at the next period, only a slight headache for a few hours was felt, and at the second period no inconvenience whatever."

From an article I wrote for the New Jersey Medical Reporter, I quote a few sentences: "The anatomical structure of the nervous system gave me a clue to the rationale of the modus operandi of dry-cupping; through the intimate connection of the spinal marrow with the whole system, for when an impression is made on the spinal marrow, it is transmitted to different organs, through the respective nerves arising from it." "Neither is there to my mind, however it may appear to others, a tithe of the incongruity in recommending dry-cupping and sponging for leucorrhea, amenorrhœa, or dysmen-orrhœa, that there is in the various plans recommended for those diseases; simply because the organ can be more easily reached and regulated through the nerves distributed to it, than by the indirect methods usually recommended." It is a fixed fact which no one will dispute, that the due performance of the function of any organ depends upon the condition of the nervous system; where that is paralyzed from any cause whatever, the organ is instantly arrested in the discharges of its peculiar function, and the derangement of the organ goes on, pari passu, with the condition of the nervous system through all the stages from total paralysis, to the slightest derangement.

With this view in our minds, a glance at the anatomy of the uterine nervous system, will render perfectly clear, why dry-cupping on the spine, is so strongly recommended in uterine affections. The uterus and its appendages are wholly supplied with nerves from the great sympathetic and sacral nerves. The right and left cords of the great sympathetic, after forming the aortic plexus, again separate, forming the right and left hypogastric nerves, which pass down giving off branches to the uterus, rectum, &c., and form a large hypogastric ganglion at the side of the cervix, behind the uterus. From the 2d, 3d and 4th sacral nerves, but chiefly from the 3d branches pass into the posterior border of the genglion at the cervix, and are lost in its mass.

From each hypogastric ganglion, numerous small nerves pass to the uterus, some of which ramify upon the muscular coat about the cervix, and others spread out under the peritoneum, to coalesce with the ganglia on the interior and posterior surface of the uterus. Large branches also accompany the uterine blood-vessels. Here we have in a nut shell, a clear explanation of the cause of the numerous sympathetic complications nearly always accompanying uterine diseases, or pregnancy; and also, a clear explanation why dry-cupping, which is a most powerful if not the most powerful alterative, so soon restores the nervous system to a healthy condition, thereby enabling the uterus to discharge its peculiar functions in a healthy manner.

We have also an explanation why dry-cupping in pregnancy, breaks the chain of nervous sympathy, and regularly applied keeps it broken, enabling a woman, free of organic disease, to go through the whole of pregnancy without scarcely any inconvenience. And, last, though not least, the distribution of the sacral nerves, gives a clear rationale, why dry-cupping on the sacrum proves so efficacious in tedious labors, as detailed in my article on the subject; partial paralysis results from it, the muscular portion of the cervix, and os uteri readily relax and a speedy delivery results, because the pains, the legitimate functions of the fundus uteri, are not interfered with in the least.

The advantages resulting from dry-cupping over the origin of the nerves, instead of the parts affected, can be concisely stated: the former strikes at the cause; the effect is felt in the part diseased, and dry-cupping on that part, strikes at the effect, instead of the cause; the former produces a powerful alterative effect, arousing every organ to full and healthy activity and is applicable to all cases; the latter produces merely temporary effects, and as Prof. Eve says, is only applicable to a certain number of cases. Why we should pay so much attention to the primæ viæ and the vascular system, neglecting the nervous system (except in neuralgic cases,) though of so much importance, I for my life cannot see any reason. While practicing in Logan county, I took charge of a case that had been in the hands of a physician for six months; as he was the best physician, I consulted him, followed the identical plan he had been pursuing, but added dry-cupping and sponging to his plan, and the result was, a speedy and permanent cure.

Those who neglect the nervous system, will no doubt think dry-cupping a great hobby with me, but of this I am confident, if they will only take a ride, they will think he rides very well. Now gentlemen, all I ask is, try Prof. Simpson's plan of dry-cupping the affected part in those cases where applicable, and in those where not applicable, try it on the spine.—*Nashville Journal of Med. & Surg.*

